# Assessing Confidence Levels in Endodontic Procedures Among Senior Undergraduate Dental Students at Qassim University, Saudi Arabia: A Cross-Sectional Observational Study

**DOI:** 10.7759/cureus.43649

**Published:** 2023-08-17

**Authors:** Abdullah Altorisy, Muhammad Zubair Ahmad

**Affiliations:** 1 Restorative Dentistry, Qassim University College of Dentistry in Ar Rass, Al Rass, SAU

**Keywords:** endodontic skill acquisition, student perception, self-assessment, clinical training, dental education

## Abstract

Background

This study aimed to determine the confidence of senior dental students in Qassim University’s undergraduate dentistry program in Saudi Arabia regarding their capacity to perform endodontic procedures. The study’s objective was to pinpoint areas of weakness and offer suggestions for potential improvement of endodontic and clinical training for dental students.

Methodology

Using anonymous survey forms, 120 senior dental students were surveyed. Students’ self-reported levels of confidence in performing various endodontic procedures, such as periapical radiographs, rubber dam placement, endodontically treated tooth restoration, anesthesia, broken instrument removal, and endodontic retreatment, were studied in this survey. A Likert-style rating scale of 1 to 5 was used in the survey, with 1 denoting high confidence and 5 denoting low confidence.

Results

The majority of senior dental students at Qassim University reported high levels of confidence in taking periapical radiographs, with 64.04% indicating a high level of confidence. The second most assured field was rubber dam installation, with 47.6% of students expressing high levels of assurance. Confidence levels were noticeably lower during more difficult procedures such as endodontic retreatment and the removal of broken instruments. While 12% of students expressed high confidence in endodontic retreatment, only 3.46% of students expressed high confidence in removing broken instruments. According to Pearson’s correlation (r = 0.352, p = 0.001), there was a significant and favorable correlation between competency level and confidence in performing various endodontic procedure-related steps.

Conclusions

The study’s findings suggest that senior dentistry students at Qassim University might benefit from additional training and assistance in some endodontic treatment areas. The lack of confidence displayed during more challenging procedures, such as the removal of broken instruments and endodontic retreatment, demonstrates the need for additional training and supervision in such cases. The results of this study may be useful for educators in other dental colleges who wish to consider developing new teaching techniques, utilizing simulators and digital learning tools, and increasing opportunities for hands-on training and supervision to improve dental students’ self-confidence and skills.

## Introduction

Dentistry is a highly diverse specialty that encompasses a multitude of branches, which students must master before graduation. Among the various branches, endodontics is considered by many students as one of the most challenging due to several factors, including patient communication, emergency situations, pain management, utilization of diverse techniques and materials, variations in root canal anatomy, interdependent steps, and the possibility of irreparable errors [1]. These factors contribute to elevated stress levels and can result in a loss of confidence among students during root canal treatment, leading to suboptimal outcomes [2]. A key factor in determining students’ abilities is the quality of their endodontic treatment, which is frequently evaluated as part of the assessment of their competency [3]. Recent assessments have shown a considerable percentage of students performing subpar endodontic operations, which raises questions about the effectiveness and caliber of their clinical training [4,5]. Students’ degree of confidence and competency in any given field is an indication of the caliber of their education, with self-confidence being crucial to their capacity to meet the predetermined goals [6,7]. Hence, a student’s ability to demonstrate their competency is largely determined by their level of self-confidence [8]. Upon graduation, it is expected that dental students be capable of accurately diagnosing and developing treatment plans for endodontic cases, as well as addressing any obstacles that may arise in the field, including referral to an endodontist when required. As the future of dentistry, it is crucial that these students receive proper encouragement and training, enabling them to become confident and successful in their practice. Through practical experience, students not only hone their skills but also provide treatments to patients [9]. A confident demeanor during procedures leads to more favorable treatment outcomes. Stress is prevalent in preclinical and clinical dental education. Teaching assistants and lecturers must know how to support students to reduce these feelings, especially during the transition period between preclinical and clinical activities. Clinical training can have a significant impact on students’ performance because they are exposed to a variety of patient-related stressors that are similar to those experienced by dentists [10,11].

At Qassim University, the clinical training and assessment of students are based on structured competencies. The competency level is assessed and graded as part of the clinical courses. This study aims to assess and analyze the confidence levels of senior undergraduate dental students at Qassim University during various stages of root canal treatment, comparing their confidence between each step and each tooth. In addition, the study seeks to identify any lack of confidence at specific stages and develop strategies, such as further training or a supportive environment, to address it, thereby fostering an environment of fear- and stress-free learning for these future practitioners of dentistry.

## Materials and methods

After obtaining ethical approval (approval number: 21-12-05) from the institutional review board of Qassim University, Saudi Arabia, we distributed anonymous electronic survey forms among the students across the following three campuses of Qassim University: Mullaydha campus (male students’ campus), Mullaydha campus (female students’ campus), and Al Rass campus on December 1, 2022. The survey has been reported and tested in the literature [5]. The survey was kept open to participants for one month. In addition to demographic questions, we included a total of 42 questions related to endodontic procedures performed by students, as well as questions related to their self-confidence levels in relation to different steps involved in procedures related to endodontic treatment.

The confidence levels in relation to the following steps of endodontic treatment were evaluated: performing anesthesia, rubber dam placement, root canal negotiation, using rotary instruments, taking periapical radiographs, removal of separated instruments, endodontic retreatment, obturation, irrigation, and restoration of endodontically treated teeth. Confidence levels of endodontic procedures among different groups of teeth, including maxillary and mandibular molars, premolars, and anterior teeth, were assessed.

We used a Likert scoring system ranging from 1 to 5, with 1 corresponding to very confident and 5 corresponding to not confident at all. The internal consistency of the questionnaire was tested (Cronbach’s alpha = 0.96).

## Results

Of the 120 students, 104 completed the survey (response rate of 86.67%). Overall, 68 were male students (65.4%), and 36 were female students (34.6%).

Table 1 describes the confidence percentage related to different steps of endodontic treatment, and Table 2 describes the confidence percentage among students related to various endodontic procedures in different groups of teeth.

**Table 1:** Confidence levels related to the steps of endodontic treatment.

Performing anesthesia	N	%
Not confident	1.5	1.44
Slightly confident	5.5	5.29
Neutral	35.5	34.13
Confident	23	22.12
Very confident	38.5	37.02
Placement of rubber dam		
Not confident	1.5	1.44
Slightly confident	5	4.81
Neutral	19.5	18.75
Confident	28.5	27.4
Very confident	49.5	47.6
Finding root canal orifices		
Not confident	5.25	5.05
Slightly confident	11.5	11.06
Neutral	33.25	31.97
Confident	32	30.77
Very confident	22	21.15
Using rotary instruments		
Not confident	10.25	9.86
Slightly confident	17.75	17.07
Neutral	43	41.35
Confident	18	17.31
Very confident	15	14.42
Taking periapical radiograph		
Not confident	1.6	1.54
Slightly confident	2	1.92
Neutral	11.4	10.96
Confident	22.4	21.54
Very confident	66.6	64.04
Removal of broken instruments		
Not confident	28	26.92
Slightly confident	19.6	18.85
Neutral	38.8	37.31
Confident	14	13.46
Very confident	3.6	3.46
Endodontic retreatment		
Not confident	12.4	11.92
Slightly confident	17.4	16.73
Neutral	30.4	29.23
Confident	26.2	25.19
Very confident	17.6	16.92
Endodontic obturation		
Not confident	4	3.85
Slightly confident	6.2	5.96
Neutral	33.6	32.31
Confident	29.8	28.65
Very confident	30.4	29.23
Endodontic irrigation		
Not confident	4	3.85
Slightly confident	10	9.62
Neutral	20.6	19.81
Confident	29.2	28.08
Very confident	40.2	38.65
Restoration of endodontically treated teeth		
Not confident	2.2	2.12
Slightly confident	3.4	3.27
Neutral	24.2	23.27
Confident	32.8	31.54
Very confident	41.4	39.81

**Table 2:** Confidence levels of endodontic procedures among different groups of teeth.

Performing anesthesia	Confident, N (%)	Not confident, N (%)
Maxillary molars	31.5 (30.29)	72.5 (69.71)
mandibular molars	30 (28.85)	74 (71.15)
Placement of rubber dam		
Maxillary molars	37.5 (36.06)	66.5 (63.94)
mandibular molars	40.5 (38.94)	63.5 (61.06)
Finding root canal orifices		
Maxillary molars	16.5 (15.87)	87.5 (84.5)
Mandibular molars	17.5 (16.83)	86.5 (83.17)
Maxillary premolars	37.5 (36.06)	66.5 (63.94)
Mandibular premolars	36.5 (35.10)	67.5 (64.90)
Using rotary instruments		
Maxillary molars	12 (11.54)	92 (88.46)
Mandibular molars	14.5 (13.94)	89.5 (86.06)
Maxillary premolars	20 (19.23)	84 (80.77)
Mandibular premolars	19.5 (18.75)	84.5 (81.25)
Taking periapical radiograph		
Maxillary molars	43 (41.35)	61 (58.65)
Mandibular molars	43 (41.35)	61 (58.65)
Maxillary premolars	45 (43.27)	59 (56.73)
Mandibular premolars	44 (42.31)	60 (57.69)
Anterior teeth	47.5 (45.67)	56.5 (54.33)
Removal of broken instrument		
Maxillary molars	9.5 (9.13)	94.5 (90.87)
Mandibular molars	8 (7.69)	96 (92.31)
Maxillary premolars	27.5 (26.44)	76.5 (73.56)
Mandibular premolars	7 (6.73)	97 (93.27)
Anterior teeth	14 (13.46)	90 (86.54)
Endodontic retreatment		
Maxillary molars	14.5 (13.94)	89.5 (86.06)
Mandibular molars	16.5 (15.87)	87.5 (84.13)
Maxillary premolars	23 (22.12)	81 (77.88)
Mandibular premolars	25.5 (24.52)	78.5 (75.48)
Anterior teeth	30 (28.85)	74 (71.15)
Obturation of root canals		
Maxillary molars	24 (23.08)	80 (76.92)
Mandibular molars	25 (24.04)	79 (75.96)
Maxillary premolars	30.5 (29.33)	73.5 (70.67)
Mandibular premolars	32.5 (31.25)	71.5 (68.75)
Anterior teeth	38.5 (37.02)	65.5 (62.98)
Irrigation of root canals		
Maxillary molars	29.5 (28.37)	74.5 (71.63)
Mandibular molars	30 (28.85)	74 (71.15)
Maxillary premolars	38.5 (37.02)	65.5 (62.98)
Mandibular premolars	36.5 (35.10)	67.5 (64.90)
Anterior teeth	39 (37.50)	65 (62.50)
Restoration of endodontically treated teeth		
Maxillary molars	36 (34.62)	68 (65.38)
Mandibular molars	35 (33.65)	69 (66.35)
Maxillary premolars	39.5 (37.98)	64.5 (62.02)
Mandibular premolars	38 (36.54)	66 (63.46)
Anterior teeth	37 (35.58)	67 (64.42)

Only 3.46% of students were very confident in the procedures of separated instrument removal. Students were most confident (64.04%) in taking periapical radiographs, followed by rubber dam placement (47.6%).

A Pearson’s correlation test was used to examine the relationship between competency level and levels of confidence in the various endodontic procedure steps, and the results revealed a significant and positive correlation between advanced competency level and high levels of confidence (r = 0.352, p = 0.001).

## Discussion

In this study, we aimed to focus on the challenges faced by senior dentistry students when performing root canal procedures. It is essential to continually assess the curriculum in the context of dentistry education and get student feedback. This aids in identifying weak points and implementing the required adjustments to raise the standard of instruction delivered [10].

We used student questionnaires as our main method of data collection to learn more about the difficulties faced by senior dentistry students. These questionnaires yielded insightful information about the students’ educational needs, which can help guide the creation of curricula [12].

The dental college in this study offers a thorough curriculum that integrates clinical practice with theoretical understanding. The fifth year of the undergraduate program, which is the final year before students enter independent clinical practice, puts considerable emphasis on the curriculum’s clinical component. The college uses an integrated clinical system that assigns each student a patient and provides them full responsibility for their dental care. This system gives students the chance to hone their clinical abilities and get practical experience while also emphasizing accountability and responsibility.

Senior dental students are required to undertake endodontic procedures as part of their advanced clinical competence in addition to general clinical experience. The goal of the curriculum creation process is to improve the standard of endodontic care delivered by senior dental students, which is accomplished in a number of ways. They included enhancing the tools and resources available for education, offering more instruction and assistance, and putting policies in place to ensure that students are held to a high standard of performance.

This study emphasizes the overall significance of ongoing assessment and feedback in dental education related to endodontics. The dental college may be able to offer students a more thorough and effective educational experience, which would increase the quality of treatment given to patients, by using student surveys and applying adjustments to the curriculum.

The study received 86.67% of the questionnaire responses, which was considered adequate to produce useful data. According to previous studies, response rates to questionnaires ranged from 47% to 100%, which might be influenced by factors such as the distribution method (e.g., email, letter, classroom, clinic) [2,6,13]. In this study, participants were urged to respond as soon as it was convenient for them to do so after receiving the questionnaires electronically during their clinical duty hours. Participants may be less likely to reply when questionnaires are distributed outside of a clinical environment, such as through email or postal mail, leading to a lower response rate. The clinical setting, however, might encourage a higher level of participant engagement and contribution readiness [14].

The students were asked to respond to a total of 42 questions (Appendices) and record their level of confidence in performing various procedures related to steps of endodontic treatment in different groups of teeth. The students recorded their confidence levels on a difficulty scale of 1-5, with 1 being the most confident and 5 being not confident at all.

While summarizing the study’s findings, it was found that molars were the most challenging group of teeth for all endodontic treatment processes. This is not a surprise as numerous studies indicate that molar endodontics is the most challenging treatment for students [13,15-17].

Maxillary molars can present significant challenges for endodontic treatment due to their physical characteristics and positioning, which can make them difficult to access. This study revealed that endodontic procedures such as retreatment, locating the canal orifices, and application of the rubber dam were particularly challenging for maxillary molars. Additionally, 71.15% of the students reported that performing local anesthesia for lower molars was also difficult, which is consistent with previous research [18,19]. In fact, studies have reported failure rates of inferior alveolar nerve block anesthesia ranging up to 85% in patients presenting with irreversible pulpitis [20]. On the other hand, the use of rotary systems, filling root canals, irrigation, and restoration following root canal therapy were shown to be areas where the students demonstrated confidence.

It can be challenging to remove fractured instruments from a root canal during endodontic treatment [21]. The majority of students (96.54%), according to our study, had not yet experienced this scenario in a dental clinic setting and lacked confidence in their capacity to retrieve a separated instrument from a root canal. This is an anticipated result because postgraduate clinics, not undergraduate clinics, are frequently responsible for retrieving broken instruments [22].

This study has some limitations. Senior undergraduate dental students of Qassim University were the only participants. Future studies should include other clinical classes and postgraduate students to gather more detailed and reliable information about the clinical program. The survey might be made available to other dental schools in the interim to allow for comparisons using additional statistical analyses and the development of a strategy for improving the clinical content and curriculum. We did not follow the CROSS guidelines to report the survey [23]. The questionnaire was not applied in surveys conducted in Saudi dental colleges before. However, it was used by other researchers and reported in the literature [5].

## Conclusions

According to the results of this study, students find maxillary molars challenging for endodontic treatment and retreatment. Most students lack the confidence in removal of separated instruments from the root canal because they have not experienced this procedure which may be attributed to advancement in root canal instrumentation procedures and a few cases of instrument separations during endodontic treatment at the study center. There is an urgent need to provide adequate training about endodontic treatment in maxillary molars and procedures to manage separated instruments in the root canals under simulated conditions and on extracted teeth. To find solutions for the delivery of high-quality skills during all types of endodontic procedures, additional studies including from other dental colleges with additional statistical analyses are required to find the weak areas during endodontic treatment.

## Appendices

Questionnaire

Title: Assessing Confidence Levels in Endodontic Procedures Among Senior Undergraduate Dental Students at Qassim University, Saudi Arabia: A Cross-Sectional Observational Study

Instructions: The survey has a total of 45 items. The identity of participants will be kept confidential at all stages of this research project.

Kindly answer all the questions. The survey will take about 20 minutes to complete.

A.   Your academic year

a.     Fourth

b.     Fifth

B.    Your clinic-campus location

a.     Qassim University female dental student in Buraydha campus

b.     Qassim University male dental student in Buraydha campus

c.     Qassim University male dental student in Ar Rass

C.    Your competency level in endodontic work according to the competency sheets provided by the department

a.     Competent or Advanced

b.     Intermediate

c.     Beginner

1.     How confident are you in performing anaesthesia for endodontic treatment in maxillary molars?

a)     very confident

b)     confident

c)     neutral

d)     little confident

e)     no confident

2.     How confident are you in performing anaesthesia for endodontic treatment in mandibular molars?

a)     very confident

b)     confident

c)     neutral

d)     little confident

e)     no confident

3.     How confident are you during placement of  rubber dam for endodontics treatment in maxillary molars?

a)     very confident

b)     confident

c)     neutral

d)     little confident

e)     no confident 

4.     How confident are you during placement of  rubber dam for endodontics treatment in mandibular molars?

a)     very confident

b)     confident

c)     neutral

d)     little confident

e)     no confident

5.     How confident are you for finding root canal orifices in maxillary molars?

a)     very confident

b)     confident

c)     neutral

d)     little confident

e)     no confident

6.     How confident are you for finding root canal orifices in mandibular molars?

a)     very confident

b)     confident

c)     neutral

d)     little confident

e)     no confident

7.     How confident are you for finding root canal orifices in maxillary premolars?

a)     very confident

b)     confident

c)     neutral

d)     little confident

e)     no confident

8.     How confident are you for finding root canal orifices in mandibular premolars?

a)     very confident

b)     confident

c)     neutral

d)     little confident

e)     no confident

9.     How confident are you for using rotary instruments in maxillary molars?

a)     very confident

b)     confident

c)     neutral

d)     little confident

e)     no confident

10.   How confident are you for using rotary instruments in mandibular molars?

a)     very confident

b)     confident

c)     neutral

d)     little confident

e)     no confident

11.   How confident are you for using rotary instruments in maxillary premolars?

a)     very confident

b)     confident

c)     neutral

d)     little confident

e)     no confident

12.   How confident are you for using rotary instruments in mandibular premolars?

a)     very confident

b)     confident

c)     neutral

d)     little confident

e)     no confident

13.   How confident are you for taking periapical radiograph in maxillary molars?

a)     very confident

b)     confident

c)     neutral

d)     little confident

e)     no confident

14.   How confident are you for taking periapical radiograph in mandibular molars?

a)     very confident

b)     confident

c)     neutral

d)     little confident

e)     no confident

15.   How confident are you for taking periapical radiograph in maxillary premolars?

a)     very confident

b)     confident

c)     neutral

d)     little confident

e)     no confident

16.   How confident are you for taking periapical radiograph in mandibular premolars?

a)     very confident

b)     confident

c)     neutral

d)     little confident

e)     no confident

17.   How confident are you for taking periapical radiograph in anterior teeth?

a)     very confident

b)     confident

c)     neutral

d)     little confident

e)     no confident

18.   How confident are you during removal of the broken instruments from root canals in maxillary molars?

a)     very confident

b)     confident

c)     neutral

d)     little confident

e)     no confident

19.   How confident are you during removal of the broken instruments from root canals in mandibular molars?

a)     very confident

b)     confident

c)     neutral

d)     little confident

e)     no confident

20.   How confident are you during removal of the broken instruments from root canals in maxillary premolars?

a)     very confident

b)     confident

c)     neutral

d)     little confident

e)     no confident

21.   How confident are you during removal of the broken instruments from root canals in mandibular premolars?

a)     very confident

b)     confident

c)     neutral

d)     little confident

e)     no confident

22.   How confident are you during removal of the broken instruments from root canals in anterior teeth?

a)     very confident

b)     confident

c)     neutral

d)     little confident

e)     no confident

23.   How confident are you for endodontic re treatment in maxillary molars?

a)     very confident

b)     confident

c)     neutral

d)     little confident

e)     no confident

24.   How confident are you for endodontic re treatment in mandibular molars?

a)     very confident

b)     confident

c)     neutral

d)     little confident

e)     no confident

25.   How confident are you for endodontic re treatment in maxillary premolars?

a)     very confident

b)     confident

c)     neutral

d)     little confident

e)     no confident

26.   How confident are you for endodontic re treatment in mandibular premolars?

a)     very confident

b)     confident

c)     neutral

d)     little confident

e)     no confident

27.   How confident are you for endodontic re treatment in anterior teeth?

a)     very confident

b)     confident

c)     neutral

d)     little confident

e)     no confident

28.   How confident are you for obturation of root canals in maxillary molars?

a)     very confident

b)     confident

c)     neutral

d)     little confident

e)     no confident

29.   How confident are you for obturation of root canals in mandibular molars?

a)     very confident

b)     confident

c)     neutral

d)     little confident

e)     no confident

30.   How confident are you for obturation of root canals in maxillary premolars?

a)     very confident

b)     confident

c)     neutral

d)     little confident

e)     no confident

31.   How confident are you for obturation of root canals in mandibular premolars?

a)     very confident

b)     confident

c)     neutral

d)     little confident

e)     no confident

32.   How confident are you for obturation of root canals in anterior teeth?

a)     very confident

b)     confident

c)     neutral

d)     little confident

e)     no confident

33.   How confident are you for irrigation of root canals in maxillary molar teeth?

a)     very confident

b)     confident

c)     neutral

d)     little confident

e)     no confident

34.   How confident are you for irrigation of root canals in mandibular molar teeth?

a)     very confident

b)     confident

c)     neutral

d)     little confident

e)     no confident

35.   How confident are you for irrigation of root canals in maxillary premolar teeth?

a)     very confident

b)     confident

c)     neutral

d)     little confident

e)     no confident

36.   How confident are you for irrigation of root canals in mandibular premolar teeth?

a)     very confident

b)     confident

c)     neutral

d)     little confident

e)     no confident

37.   How confident are you for irrigation of root canals in anterior teeth?

a)     very confident

b)     confident

c)     neutral

d)     little confident

e)     no confident

38.   How confident are you for restoration of endodontically treated maxillary molar teeth?

a)     very confident

b)     confident

c)     neutral

d)     little confident

e)     no confident

39.   How confident are you for restoration of endodontically treated mandibular molar teeth?

a)     very confident

b)     confident

c)     neutral

d)     little confident

e)     no confident

40.   How confident are you for restoration of endodontically treated maxillary premolar teeth?

a)     very confident

b)     confident

c)     neutral

d)     little confident

e)     no confident

41.   How confident are you for restoration of endodontically treated mandibular premolar teeth?

a)     very confident

b)     confident

c)     neutral

d)     little confident

e)     no confident

42.   How confident are you for restoration of endodontically treated anterior teeth?

a)     very confident

b)     confident

c)     neutral

d)     little confident

e)     no confident
